# Clinicopathological features of the rare form of Creutzfeldt-Jakob disease in R208H-V129V *PRNP* carrier

**DOI:** 10.1186/s40478-019-0699-1

**Published:** 2019-03-21

**Authors:** Dorina Tiple, Anna Poleggi, Vittorio Mellina, Antonino Morocutti, Livia Brusa, Cesare Iani, Elisa Colaizzo, Luana Vaianella, Simone Baiardi, Anna Ladogana, Piero Parchi, Maurizio Pocchiari

**Affiliations:** 1 Department of Neuroscience, Istituto Superiore di Sanità, Viale Regina Elena 299, 00161 Rome, Italy; 2 Department of Neurology, S. Eugenio Hospital, Rome, Italy; 3 IRCCS Istituto delle Scienze Neurologiche di Bologna, Bologna, Italy; 4 Department of of Experimental Diagnostic and Specialty Medicine (DIMES), University of Bologna, Bologna, Italy

**Keywords:** Genetic Creutzfeldt-Jakob disease, Prion diseases, Neuropathology, R208H, Dementia, Mutation

To the Editor,

Genetic transmissible spongiform encephalopathy (TSE) diseases are always associated with one of the more than 50 disease-associated point or insert mutations of the PrP gene (*PRNP*) [12] and represent approximately 10 to 20% of all forms of TSE diseases [9]. Each mutation is often associated with specific clinic-pathological phenotype [12] that are generally represented by Creutzfeldt-Jakob disease (CJD) [3, 8], Gerstmann–Sträussler–Scheinker disease or inherited prion protein cerebral amyloidoses [5], and fatal familial insomnia [4]. The methionine/valine polymorphism at codon 129 of *PRNP* plays also a role in determining the disease phenotype, especially when co-segregates with the pathogenic mutation [3]. Most *PRNP* mutations responsible for the CJD phenotype, including the R208H, are extremely rare and often there is no evidence of CJD in other family members. In particular, the R208H mutation co-segregates either with methionine or valine at codon 129 and it has been fully described in only 12 patients carrying M129 and 4 patients with V129 [8]. Here, we report clinical and neuropathological details of the fourth worldwide case of CJD carrying the rare R208H-129 Val *PRNP* genotype with a suggestive positive family history for dementia.

## Clinical findings

A 64-year-old woman developed psychiatric symptoms with mood depression and apathy, and unsteadiness while walking in July 2003. Two months later, ataxic gait and left lateropulsion were reported. On November 2003, the patient was hospitalized and neurological examination showed reduced psychomotor ability, despite the patient was alerted and oriented, mild hypophonia, cerebellar dysarthria, mild dysmetria of the upper and lower limbs, head tremor, and tendency to retro- and latero-pulsion in Romberg position. The gait was possible only with bilateral support. The *Mini Mental State Examination* was 21.2/30.

Seven months after disease onset, the patient developed myoclonus in the perioral region and in the left hand, extrapyramidal (rigidity) and pyramidal (Babinski) signs, paratonia in all limbs, and finally akinetic mutism. EEG evolved in diffuse periodic sharp-waves complexes, which lasted until death. Cerebrospinal fluid analysis showed a positive 14–3-3 protein test. Brain MRI, on FLAIR sequences, showed a mild hyperintensity of the nucleus caudatus bilaterally and of the peri-aqueductal gray matter. Direct sequencing of the *PRNP* ORF (See details of methods in Additional file 1) revealed a point mutation at codon 208 (R208H) causing the substitution of Histidine for Arginine coupled with homozygosity for Valine at codon 129. A diagnosis of probable genetic CJD was made according to the internationally recognised diagnostic criteria [10].The patient died 30 months after onset and autopsy was performed. RT-QuIC test on CSF performed a posteriori resulted positive (See details of methods in Additional file 1) . Family members reported that the mother of the patient developed memory impairment for which she was admitted to hospital with a suspect of cerebral neoplasia and died 6 months later at age 80. Hospital records were not available. Patient’s father died at age 84 without dementia or neurological signs while her younger sister died at age 48 in a car accident. She had two healthy children. First-degree family members refused genetic examination.

## Neuropathology and immunochemistry

**Fig. 1:**
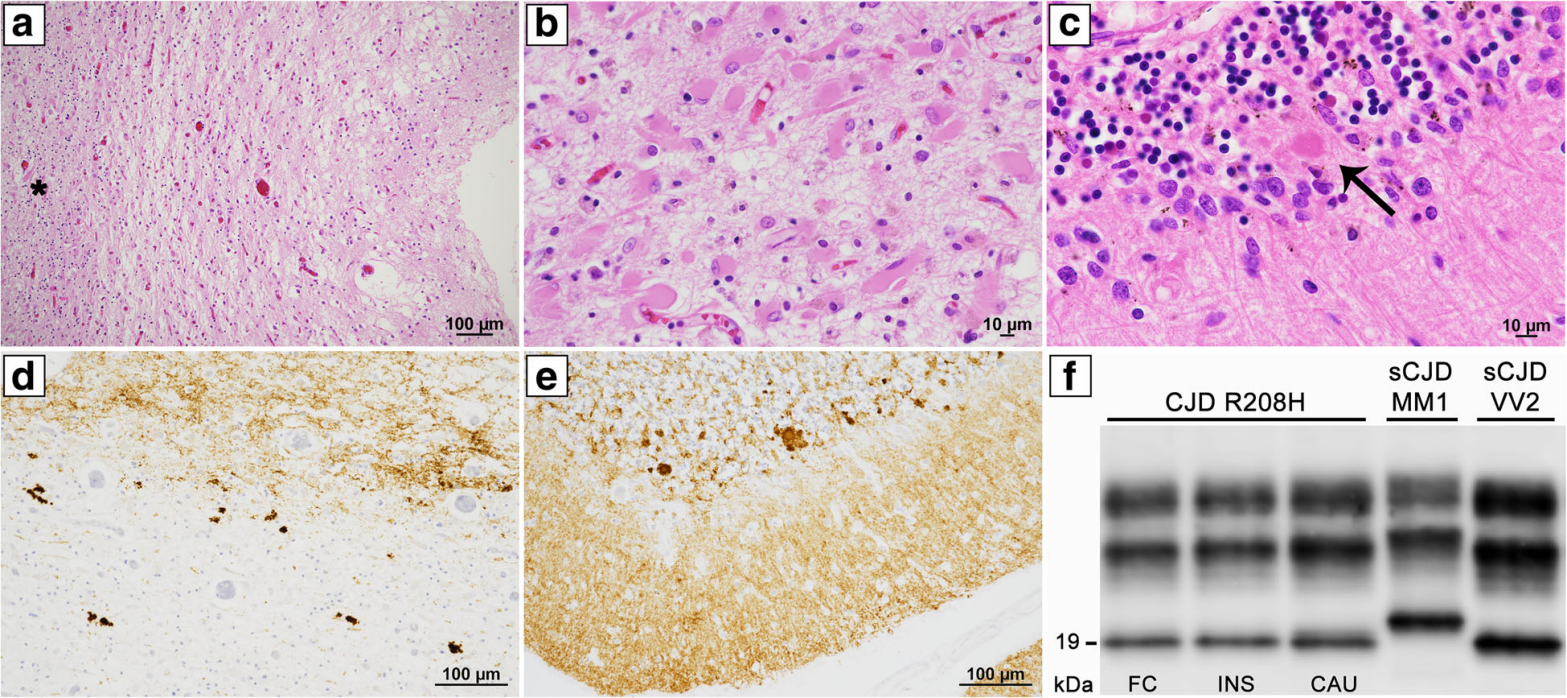
Histopathological features and western blot profile of PrP^TSE^ in CJD R208H-129V. Status spongiosus associated with severe gliosis and neuronal loss in the temporal cortex (**a**, H&E, the asterisk indicates the white matter junction). Severe gliosis of subcortical white matter with many gemistocytic astrocytes and scattered macrophages (**b**, H&E, frontal lobe). Small, unicentric amyloid plaque (arrow) at the transition between the molecular and granular layers of the cerebellum (**c**, H&E). Synaptic PrP staining in the cortical gray matter and plaque-like deposits in subcortical white matter (**d**, PrP immunohistochemistry, temporal lobe). Diffuse synaptic pattern of PrP^TSE^ deposition in the molecular layer and plaque-like deposits associated with few small plaques in the granular layer of the cerebellum (**e**, PrP immunohistochemistry). Immunoblot profiles of PK-treated PrP^TSE^ in CJD R208H, sCJD MM1 and sCJD VV2 (**f**). Samples were probed with the primary antibody 3F4. Relative molecular masses are expressed in kDa. FC: frontal cortex, INS: insular cortex, CAU: caudate nucleus

Gross examination of the brain showed severe diffuse atrophy of the cerebral hemispheres and cerebellum. Microscopic examination showed status spongiosus associated with severe gliosis, and loss of neurons in the cerebral cortex of all lobes (Fig. 1a, see details of methods in Additional file 1), striatum, thalamus, and cerebellum. Moderate to severe gliosis and neuronal loss also affected the subiculum, substantia nigra, brainstem periaqueductal gray and the inferior olive. The hippocampal formation (CA2-CA4) was relatively spared and uniquely showed foci of classic spongiform change.

Severe demyelination and axonal loss, accompanied by severe gliosis including many gemistocytic astrocytic and scattered macrophages diffusely affected the subcortical white matter (Fig. 1b). Sparse small, unicentric amyloid plaques were seen in the cerebellar granular layer (Fig. 1c); however classic mature and fully formed plaques of the kuru type were rare. Overall, the number of amyloid plaques were significantly lower compared to those seen in sporadic CJD MV2K with comparable disease duration (mean of 3 cases).

PrP immunohistochemistry demonstrated a diffuse synaptic pattern of PrP^TSE^ in the cerebral cortex and scattered small focal plaque-like deposits in the subcortical white matter immediately below the cortical ribbon (Fig. 1d), in the granular layer of the cerebellum (Fig. 1e) and the cerebellar white matter. Immunostaining for phospho-tau, amyloid-beta, alpha-synuclein, and phospho-TDP-43 revealed no abnormal protein deposits (See details of methods in Additional file 1) .

PrP^TSE^ was detected in all areas analyzed. The physicochemical properties of the abnormal protein were consistent with that of PrP^TSE^ type 2A from sporadic CJDVV2 with an unglycosylated form migrating at 19 kDa. PrP ^TSE^ monoglycosylated and diglycosylated bands were either even or showed a predominance of the monoglycosylated form as in sporadic CJDVV2 (Fig. 1f, see details of methods in Additional file 1).

The R208H mutation coupled with V129 have been so far described only in 4 unrelated CJD patients in three European countries (France, Czech Republic, and Italy) (Table 1), contrasting with a wider geographical distribution of R208H mutation coupled with M129 [8]. The case described here, similarly to the previously described ones [2, 11, 13], clinically resembles codon 129VV sporadic CJD for clinical and neuropathological features. However, behavioural changes are more frequently present at onset in R208H V129 (75%) compared with VV sporadic CJD patients (7.5%) and disease duration, especially in the case reported here, is particularly long [1]. The most helpful diagnostic features is brain MRI that shows the characteristic hyperintensity in subcortical and cortical areas [14] in 3 of 4 cases. The result of the RT-QuIC test in the CSF resulted positive in this case but negative in the other previously described Italian case [13] suggesting caution in the interpretation of negative RT-QuIC in carriers of CJD patients with the R208H V129 mutation. The comparison of brain pathology in the autopsied cases revealed plaque-like structures seen with immunohistological investigation in all three cases in line with similar staining in sporadic CJD with type 2 PrP^TSE^ and valine homozygosity [1], while the presence of true amyloid Kuru plaques as in the case of Basset-Leobon and colleagues [2] is not observed in the same molecular subtype of sporadic CJD. However, the brain histology of our case differed significantly because of the presence of status spongiosus and severe astrogliosis that affected white matter, with a picture very similar to that reported in the panencephalopatic form of CJD [7] unlikely dependent by the R208H mutation but in line with long disease duration [6]. Finally, the case reported here differs from previously described cases because of the presence of a rapid (6 months) cognitive decline in the mother’s patient that might suggest CJD.

**Table 1:** Characteristics of CJD patients carrying the R208H-129 VV haplotype

		CJD cases^a^			
		1	2	3	4
Country		France	Czech Republic	Italy	Italy
Sex/Age at onset (years)		M/61	F/62	F/62	F/63
Family history for CJD/dementia		No/No	No/No	No/No	No/Yes
Disease duration (months)		7	16	9	30
Symptom at onset		Psychiatric symptoms	Lower back pain, gait disturbances	Akinesia, postural and psychiatric symptoms	Psychiatric symptoms
Clinical signs	Cognitive impairment	Yes	Yes	Yes	Yes
	Cerebellar	Yes	No	No	Yes
	Visual	No	No	No	No
	Pyramidal/Extrapyramidal	No	Yes	Yes	Yes
	Myoclonus	No	Yes	No	Yes
	Akinetic mutism	Yes	No	Yes	Yes
Diagnostic features (months from onset)	MMSE	Not reported	29/30 (8) 17/30 (10)	12/30 (3)	21/30 (4)
	EEG typical for CJD^b^	No (5)	Yes (8)	No (5)	Yes (7)
	Brain MRI typical for CJD^c^	No (5)	Yes (8)	Yes (4)	Yes (4)
	14-3-3 in CSF	Negative (5)	Positive (10)	Negative (6)	Positive (7)
	RT-QuIC in the CSF	Not done	Not done	Negative (6)	Positive (7)
PrP^CJD^ type		Type 2A	Type 2A	Not done	Type 2A
Neuropathology		Spongiform changes in frontal cortex and striatum; gliosis in striatum and thalamus; Kuru plaques in cerebellum	Spongiform changes in frontal cortex; plaque-like structures in cerebral and cerebellar cortices, and basal ganglia	Not done	Status spongiosus in cerebral cortex, striatum, thalamus and cerebellum; severe astrogliosis in white matter; unicentric amyloid plaques in the cerebellum

^a^Case 1, Basset-Leobon et al. [2]; case 2, Matej et al. [11]; case 3, Vita et al. [13]; case 4, reported here

^b^Generalised triphasic periodic complexes

^c^High signal in caudate/putamen on MRI brain scan or at least two cortical regions (temporal, parietal, occipital) either on DWI or FLAIR

## Additional file

Additional file 1: Supplementary data. (DOCX 21 kb)

## Abbreviations

CJD: Creutzfeldt-Jakob disease
CSF: Cerebrospinal fluid
EEG: Electroenchephalogram
FLAIR: Fluid attenuated inversion recovery
MRI: Magnetic resonance imaging
ORF: Open reading frame
*PRNP*: Human prion protein gene
PrP: Prion protein
PrP^TSE^: Pathological prion protein
RT-QuIC: Real time quake induced conversion
TSE: Transmissible spongiform encephalopathy
