# Supplementary material for: Clinicopathological features of the rare form of Creutzfeldt-Jakob disease in R208H-V129V PRNP carrier
Source: Acta Neuropathol Commun. 2019 Mar 21;7:47. doi: 10.1186/s40478-019-0699-1 (PMC6429782; doi:10.1186/s40478-019-0699-1)
Supplement: Supplementary file 1 — Supplementary data. (DOCX 21 kb) [file 40478_2019_699_MOESM1_ESM.docx]

**Supplementary data**

**Methods**

*Molecular genetic analysis.* After an informing consent was signed blood samples were collected and genomic DNA was extracted from peripheral leukocytes according to standard procedures. The *PRNP* open reading frame (ORF) was amplified by the PCR with the flanking primers PrPF (5'-GATACCATTGCTATGCACTCATTC-3') and PrPR (5'-CATCAGTGCCAAGGGTATTGATTAGCC-3') in an automated thermocycler T100 (Bio-Rad). PCR was run through 40 cycles at 94 °C for 40 seconds, at 60 °C for 40 seconds, at 72 °C for 40 seconds, plus a final step at 72 °C for 5 minutes. PCR products were detected on 1.5% agarose gel, purified with the QIAquick PCR purification kit (Qiagen) according to manufacturer’s recommendations, and sequenced by the Sanger dideoxynucleotide chain termination method with the kit GenomeLab DTCS-Quick Start Kit (Beckman-Coulter). The PrPF, PrPR primers, and two additional internal primers, PrPRi (5'-GTCTCGGTGAAGTTCTCCCC-3') and PrPFi (5'-ACAGTCAGTGGAACAAGCCG-3'), were used. Sequences were run for 30 cycles at 96 °C for 20 seconds, 58°C for 20 seconds and 60 °C for 4 minutes. Purified sequences were then electrophoresed on the automated CEQ8000 Genetic Analysis System (Beckman-Coulter) and analyzed using the software provided by the manufacturer. The reference sequence was obtained by NCBI gene bank (<https://www.ncbi.nlm.nih.gov/gene/?term=PRNP>).

*RT-QuIC* test. The Real Time Quaking Induced Conversion (RT-QuIC) assay was performed in quadruplicate by seeding 30 μl of CSF for each sample, according to McGuire et al [1] with modifications, on a FluoStar OMEGA plate reader at 42 ◦C.

*Neuropathology*. At autopsy, the right half of the brain was immediately frozen, while the left half was fixed in formalin and used for neuropathological examination. Samples from the cerebral neocortex (two for each lobe), cingulate, entorhinal, and insular cortices, hippocampus, amygdala, striatum, thalamus (anterior and posterior), midbrain, pons, medulla and cerebellum (vermis, and hemisphere) were taken from the left half of the brain and used for histopathologic examination. A semiquantitative evaluation of spongiform change, neuronal loss and gliosis was carried out on H&E stained paraffin sections. Immunohistochemistry for PrP (3F4) and proteins phospho-tau (AT8), beta-amyloid (4G8), alpha-synuclein (LB509) and phospho-TDP-43 (pS409/410-1) was performed as described [2]. Selected sections from the cerebral cortex and cerebellum were also stained with PAS, and Congo red. Astrocytic gliosis and white matter pathology were also assessed on slides stained with anti- GFAP (Dako), HLA-DR-DQ (Dako), and APP (Chemicon) monoclonal antibodies.

*Biochemical analysis*. PrP^TSE^ was extracted from the above-listed brain regions of the right side. Immunoblot analysis of PrP^TSE^ was carried out as described [3], using the monoclonal antibodies 3F4 (1:40000), which recognize the human PrP residues 109-112.

**References**

1.McGuire LI, Poleggi A, Poggiolini I, Suardi S, Grznarova K, Shi S et al (2016) Cerebrospinal fluid real-time quaking-induced conversion is a robust and reliable test for sporadic Creutzfeldt-Jakob disease: An international study. Ann Neurol 80:160-5

2.Rossi M, Saverioni D, Di Bari M, Baiardi S, Lemstra AW, Pirisinu L, et al (2017) Atypical Creutzfeldt-Jakob disease with PrP-amyloid plaques in white matter: molecular characterization and transmission to bank voles show the M1 strain signature. Acta Neuropathol Commun 5:87.

3.Parchi P, Notari S, Weber P, Schimmel H, Budka H, Ferrer I et al (2009) Inter-laboratory assessment of PrPSc typing in Creutzfeldt-Jakob disease: a Western blot study within the NeuroPrion Consortium. Brain Pathol 19:384-91
